# Mental health and well-being of children in the Philippine setting during the COVID-19 pandemic

**DOI:** 10.34172/hpp.2021.34

**Published:** 2021-08-18

**Authors:** Grace Zurielle C. Malolos, Maria Beatriz C. Baron, Faith Ann J. Apat, Hannah Andrea A. Sagsagat, Pamela Bianca M. Pasco, Emma Teresa Carmela L. Aportadera, Roland Joseph D. Tan, Angelica Joyce Gacutno-Evardone, Don Eliseo Lucero-Prisno III

**Affiliations:** ^1^College of Medicine, University of the Philippines Manila, Manila, Philippines; ^2^Matias H. Aznar Memorial College of Medicine, Cebu City, Philippines; ^3^West Visayas State University-College of Medicine, La Paz, Iloilo City, Philippines; ^4^Faculty of Medicine and Surgery, University of Santo Tomas, Manila, Philippines; ^5^Baguio General Hospital and Medical Center, Baguio City, Philippines; ^6^Department of Pediatrics, Eastern Visayas Regional Medical Center, Tacloban City, Philippines; ^7^Faculty of Management and Development Studies, University of the Philippines Open University, Los Banos, Laguna, Philippines; ^8^Department of Global Health and Development, London School of Hygiene and Tropical Medicine, London, United Kingdom

**Keywords:** Mental Health, Philippines, COVID-19, Psychology, Child, Child care, Health services, Social problems

## Abstract

The coronavirus disease 2019 (COVID-19) pandemic has subjected the mental health and well-being of Filipino children under drastic conditions. While children are more vulnerable to these detriments, there remains the absence of unified and comprehensive strategies in mitigating the deterioration of the mental health of Filipino children. Existing interventions focus on more general solutions that fail to acknowledge the circumstances that a Filipino child is subjected under. Moreover, these strategies also fail to address the multilayered issues faced by a lower-middle-income country, such as the Philippines. As the mental well-being of Filipino children continues to be neglected, a subsequent and enduring mental health epidemic can only be expected for years to come.

## Introduction

The Philippine Development Plan for 2017-2023 highlights that children are among the most vulnerable population groups in society, including them in strategies for risk reduction and adaptive capacity strengthening.^1^ Approximately 40% of the total Philippine population is comprised of Filipinos below 18 years of age.^2^ Despite having a large portion of the Philippine population declared as vulnerable, concerning issues involving them still persist and remain unaddressed.

Among Filipino children aged 5 to 15, 10% to 15% are affected by mental health problems.^3^ According to the World Health Organization (WHO), 16.8% of Filipino students aged 13 to 17 have attempted suicide at least once within a year before the 2015 Global School-based Student Health survey.^4^ This is just one of the many indicators showing the state of mental health of these children. These statistics involving children’s mental health are concerning as childhood is a crucial period where most mental health disorders begin. Efforts should be made to identify these issues early for proper treatment in prevention of negative health and social outcomes.^4^ Childhood mental and developmental disorders also frequently persist into adulthood, making it more likely for them to have compromised growth with greater need for medical and disability services and higher risk of getting involved with law enforcement agencies.^5^ In this context, the COVID-19 pandemic threatens to worsen these numbers, affecting the delivery of the Philippines’ health care services, including those for children’s mental health.

Since the beginning of the pandemic, children have been subjected to multiple threats to their mental health. Adding insult to injury, several concurrent factors in the Philippine society exacerbate this. While these are experiences shared by all people regardless of age, impediments to emotional and social development are greater in children than in adults.^6^ They may also be more vulnerable to developing mental health issues such as depression and anxiety.^7^ Together with these circumstances and the weakened health care system, children’s vulnerability towards mental health problems may be worsened by the pandemic, leading to more new cases and exacerbating existing ones.^2^

## Status of mental health system for children in the Philippines

According to the National Statistics Office (NSO), mental health illnesses rank as the third most common form of morbidity among Filipinos.^8^ In the assessment conducted on the Philippine mental health system, a prevalence of 16% of mental disorders among children was reported.^9^ With this alarming number of cases, it is surprising to see how the Philippines is currently responding to this problem. To date, there are only five government hospitals with psychiatric facilities for children, 84 general hospitals with psychiatric units, and 46 outpatient facilities from which there are only 11 that are designated for children and adolescents. Additionally, there are only 60 child psychiatrists practicing in the Philippines, with the majority of them practicing in urban areas such as the National Capital Region. Hence, children with mental health problems who are in rural areas have less access to such services.^10^

As the pandemic continues, combined with the menace of the typhoon season, thousands of children are placed in a situation where the future is uncertain. A local study showed that youth age and students are among those with significant association to a greater psychological impact due to the pandemic.^11^ In addition, UNICEF also reports that children nowadays face a trifecta of threats which include direct consequences of the disease itself, interruption in essential services, and increasing poverty and inequality. All of these can lead to higher incidences of stress, anxiety, and depression.^12^

## General mental health implications of COVID-19 on Filipino children

The fear and anxiety of contracting the virus, the suspension of physical classes, the disruption of regular daily routine, and the decrease of social support from school peers collectively add burden to the mental well-being of children.^7,13^ The shift to online classes increases the burden on the mental well-being of children. Excessive use of these technologies has been associated with developmental delays and has resulted in sleep schedule disruptions.^14^ This situation is aggravated by the strict implementation of the confinement of children at home. Children living with preexisting mental health concerns,^13^ and living in cramped households and communities face worse circumstances.

## Militarization of the Philippine COVID-19 response

Aside from being regarded as one of the countries with the longest lockdown, the Philippines has also been called out by the United Nations for employing a highly militaristic approach in response to the COVID-19 pandemic.^15^Militarization may come across as threatening, because it implies a potential for violence.^16^ Furthermore, few studies abroad have reported that children and adolescents may tend to view police forces as punitive figures whom they fear.^17,18^ While these qualitative studies were conducted long before the current health crisis began, it may be possible for increased military presence in communities to exacerbate the fears already emanating from the pandemic itself; this can negatively impact a child’s psychological development.^4^ Still, local evidence to confirm these associations, especially in the context of the pandemic, is lacking. Many studies have already documented the impact of lockdown on children, but none of them have looked into how the strategies for implementation may also be contributory to their mental health or well-being.

## Typhoons and the mental health of Filipino children

The Philippines has been hit by 22 tropical typhoons during the COVID-19 pandemic, leaving thousands of families homeless.^19^ Children who are already frightened of COVID-19 and previous tropical storms have had to relive their experience with each new typhoon that came. In addition, children in crowded evacuation centers are at increased risk of contracting diseases and experiencing gender-based violence.^20^ Given how past typhoons of similar strength and destruction have caused lasting adverse mental health effects on children,^21^ the same or even worse, may be expected as a result of the more recent calamities. Super typhoons *Goni* and *Vamco* have caused further disruptions in schooling and livelihood, therefore leaving more children vulnerable to the effects of the pandemic. Those who have been forced to seek refuge in evacuation centers are at an increased risk of acquiring COVID-19, among other diseases.^20^

## Child Labor and Abuse in the Time of COVID-19

The COVID-19 crisis caused an unprecedented reduction in economic activity and working time, thus increasing poverty. Fewer employment opportunities and lower wages drive exploitative work. Further suppression of wages induces child labor. There may be deliberate recruitment of children to cut costs and boost earnings.^22^

In addition to the threats of child labor, a study entitled The Hidden Impact of COVID-19 on Children reported that violence occurred in nearly one-third (32%) of households. Lesser household incomes were associated with more reports of violence towards children.^23^ According to UNICEF, the Philippine government saw a 260% increase in online child abuse reports from March-May. Many victims are first abused by their parents, who livestream sexual violence for predators in wealthy Western nations. This occurrence resulted from job and income loss and more time spent at home due to strict quarantine measures. The abuse in children occurs at an average of 2 years before being rescued.^24^

## Strategies Addressing the mental health implications of COVID-19 on Filipino children

Numerous strategies have been utilized to address the mental health impacts of COVID-19 on Filipinos. With the mental health implications predicted at the beginning of the pandemic, the Psychological Association of the Philippines has compiled a list of free telemedicine consultations. As of August 24, the Philippine Red Cross has also established a COVID-19 hotline with 9790 helpline volunteers to address mental health and other similar concerns. The Department of Health has also conducted nationwide campaigns in observance of the National Mental Health Week.^25^

Albeit present, these interventions are limited to the general population, and strategies specific to addressing the mental health situation of children remain scarce and staggered. Compounding factors of classifying among the lower- to middle-income countries of militarization, natural disasters, and child labor and abuse have yet to be considered. In addition, it is also important to consider that happiness, with its multifactorial nature, is a vital component of an individual’s overall wellbeing.^26^

## Conclusion

The already-challenged state of mental well-being of Filipino children has been worsened by the pandemic and the lack of good mental health policies by the government. While there is increasing awareness for mental health, children-centered interventions remain deficient. Approaches must integrate commonly-known mental health effects on children with existing and anticipated Philippine societal issues. Without doing so, it may be expected that as the COVID-19 pandemic is mitigated, a mental health epidemic will replace it.

## Funding

None.

## Competing interests

The authors have no conflicts of interest.

## Ethical approval

Not applicable.

## Authors’ contributions

GZCM and DELP were involved in the conception of the paper. GZCM led the writing of the manuscript and acted as corresponding author. GZCM, MBCB, FAJA, HAAS, PBMB, ETCA and RJDT wrote sections of the manuscript. AJGE and DELP reviewed and edited the initial draft of the manuscript prior to submission. All authors have reviewed and agreed to the final version of the paper.
